# Effect of Hulless Barley Flours on Dough Rheological Properties, Baking Quality, and Starch Digestibility of Wheat Bread

**DOI:** 10.3389/fnut.2021.785847

**Published:** 2021-12-13

**Authors:** Liwei Yu, Yanrong Ma, Yiyue Zhao, Yilin Pan, Renmei Tian, Xiaohua Yao, Youhua Yao, Xinyou Cao, La Geng, Zhonghua Wang, Kunlun Wu, Xin Gao

**Affiliations:** ^1^State Key Laboratory of Plateau Ecology and Agronomy, Qinghai Key Laboratory of Hulless Barley Genetics and Breeding, Qinghai Subcenter of National Hulless Barley Improvement, Qinghai University, Xining, China; ^2^State Key Laboratory of Crop Stress Biology in Arid Areas and College of Agronomy, Northwest A&F University, Xianyang, China; ^3^Shandong Academy of Agricultural Sciences/National Engineering Laboratory for Wheat and Maize/Key Laboratory of Wheat Biology and Genetic Improvement in North Yellow and Huai River Valley, Crop Research Institute, Ministry of Agriculture, Jinan, China; ^4^Institute of Crop Science, Zhejiang University, Hangzhou, China

**Keywords:** hulless barley, wheat bread, nutritional function, *in vitr*o digestibility, dough mixing properties

## Abstract

Hulless barley (*Hordeum vulgare* L.), also known as highland barley, contains nutritional compounds, such as β-glucan and polyphenol, which can be added to wheat flour to improve the dough nutritional quality. In this study, different formulated dough samples were obtained by individually adding four hulless barley flours into flour of a wheat variety (Jimai 44, designated as JM) which has very strong gluten. The effects of hulless barley supplementation on gluten structure, dough rheological properties, bread-making properties, and starch digestibility were assessed. The results showed that compared with JM dough, substitution of hulless barley flour to wheat flour at levels ranging from 10 to 40% negatively affected gluten micro-structure and dough mixing behavior, because the cross-links of gluten network were partially broken and the dough development time and stability time were shortened. For the hulless barley-supplemented bread, specific volume was significantly (*P* < 0.05) increased while springiness was not greatly changed. Furthermore, the hydrolysed starch rate in hulless barley-supplemented bread was decreased, compared with that in JM bread. Importantly, the contents of β-glucan, polyphenols and flavonoids in hulless barley-supplemented bread were 132.61–160.87%, 5.71–48.57%, and 25–293.75% higher than those in JM bread, respectively. Taken together, the hulless barley-supplemented bread has been fortified with enhanced nutritional components, more desirable bread-making quality, and improved starch hydrolytic properties, which shows a great potential to use hulless barley as a health supplement.

## Introduction

Bread is one of the staple foods to provide nutrition. However, most bread made from fine wheat flour lacks nutrients such as vitamins, minerals, and dietary fiber (1, 2). Besides, it contains a high content of rapidly digestible starch which increases postprandial glucose level, and therefore is not suitable for diabetic patients (3). For these reasons, some novel bread formulas, which can improve nutritional quality and reduce starch digestion rate, are favored by consumers.

Hulless barley is a kind of cereal originated from the Qinghai-Tibet Plateau in China, which has strong adaptability to some extremely inhospitable environments in high altitude areas (4). The morphological characteristics and features of hulless barley are similar to those of wheat, while it contains more healthy nutrients than wheat does, such as dietary fiber (11.70–12.96%), β-glucan (4.4–5.5%), polyphenol (2.71–4.37 mg·g^−1^), and flavonoids (0.56–2.86 mg·g^−1^) (5, 6). A recent study showed that white rice supplemented with 50% high β-glucan (7.2%) barley can inhibit postprandial blood glucose and insulin levels in patients with type 2 diabetes (7). Intraperitoneal injection of β-glucan (400 and 800 mg/kg/day) could substantially reduce the mortality of X-ray whole-body irradiated mice and the tumor growth of tumor-bearing mice (8). In addition, polyphenols in hulless barley can prevent oxidation, and intake of total flavonoids is associated with a lower risk of death from coronary heart disease (*P* = 0.04) (6, 9, 10). In recent years, due to the health benefits of hulless barley, the research of hulless barley as a functional food has aroused great interest among researchers (11, 12). However, the poor sensory quality of hulless barley, coupled with its poor baking quality, limits its use in food industry (11). Therefore, developing new products with high nutritional value by adding hulless barley to wheat bread can not only meet the demand for health food, but also promote applications of hulless barley in food industry. Thus, the bread supplemented with hulless barley needs to be investigated. So far, a few studies have investigated the quality characteristics of hulless barley and found that quality properties vary among different varieties of hulless barley (4–6). Among these studies, most researchers have focused on the effect of substitution level of hulless barley on properties of wheat dough, but there have been few reports on wheat dough supplemented with different varieties of hulless barley. No study has used different varieties and levels of hulless barley to substitute wheat flour and investigated the factors that affect bread making quality and health benefits.

Wheat dough can be characterized by rheological (storage and loss modulus) and mixing indices (development time and stability time of dough), which are crucial characteristics closely related to the processing quality of dough and thus the quality of the final products (13, 14). The dough processing quality has been perceived to be greatly affected by the composition and structure of gluten protein (13). Wheat gluten is divided into gliadins and glutenins, and the ratio of glutenins to gliadins (Glu/Gli) has been usually used to reflect viscoelasticity of dough (15, 16). Glutenins are composed of high- and low-molecular-weight glutenin subunits (HMW-GS and LMW-GS), and the ratio of HMW-GS to LMW-GS (H/L) has been adopted to determine dough stability (15, 16). Moreover, it has been reported that the percentage of unextractable polymeric protein in total polymeric protein (UPP%) in wheat grain positively correlates with the dough stability (14). The micro-structure of gluten can be quantitatively characterized by lacunarity, protein area and total protein length, and has been reported to significantly affect dough stability (14, 17). Previous studies on gluten structure showed that supplementation of plant materials destroys the protein skeleton of wheat dough, resulting in discontinuous gluten networks, which further affects the quality of final products (10, 18). However, there has been no report on the effects of hulless barley on protein composition of wheat dough. Therefore, the investigation of Glu/Gli ratio, H/L ratio and UPP% of hulless barley supplemented wheat flours provides insight into gluten characteristics of dough formulations, and can also provide valuable information on protein quality traits of hulless barley for future research.

Starch, another main component of dough, has an important effect on the quality of final products (19, 20). Considering the differences in the starch physicochemical properties between hulless barley and wheat (4), when wheat and hulless barley flours are mixed for bread making, great changes are expected to take place in starch gelatinization, which further affect the processability of dough. Starch granules can be divided into A-type (diameter more than 10 μm) and B-type (diameter no more than 10 μm), whose size distribution has been reported to affect the processing quality of wheat dough (21), and B granules have been suggested to have a positive contribution to the stability of dough (14). On the other hand, the size and type of starch granules, and amylose content have been perceived to affect the digestibility of starch (22). It has also been suggested that foods with high amylose content are preferable for diabetic patients (23). Therefore, it is of significance to clarify the starch properties of formulated flours for effective processing in bread production.

This study used a wheat variety (Jimai 44) with very strong gluten and four hulless barley varieties with significant differences in quality characteristics and nutritional composition as experimental materials, to investigate the effects of hulless barley on processing quality and nutritional function of wheat bread. The gluten structure and rheological properties of the flour formulations were investigated, and the textural properties and starch digestibility of the bread were analyzed. The results of the current study can provide useful information on processing and applications of hulless barley supplemented flours as a health food.

## Materials and Methods

### Materials

The experimental materials included Jimai 44 (JM), one wheat variety with very strong gluten and four hulless barley varieties namely Heilaoya (HB1), Kunlun 14 (HB2), Kunlun 15 (HB3), and Kunlun 17 (HB4). Field experiments were conducted in Yangling, China (108°4′E, 34°16′N) during the growing seasons of 2018–2019 and 2019–2020. The harvested grains were ground in a Brabender Quadrumat Senior (Brabender Instruments, New Jersey, USA) and sieved (100 mesh) to obtain refined wheat and hulless barley flours.

Wheat flour and hulless barley flours were mixed into formulations before analysis, with different ratios of wheat flour to hulless barley flour by weight (90:10, 80:20, 70:30, and 60:40). A 100% JM wheat flour sample was used as a control.

### Analyses of Grain Quality Characteristics

Grain quality-related parameters were measured by the wheat grain model and hulless barley model, respectively, on a near-infrared reflectance (NIR) spectrum instrument (Diode Array 7250 Perten, Huddinge, Sweden) according to Miralbés (24), for characteristics such as moisture, protein content, starch content and total dietary fiber content in grains of the wheat variety and the four hulless barley varieties. The measurements were carried out in three individual replicates.

### Determination of Glu/Gli Ratio, H/L Ratio, and UPP%

Gliadins and glutenins were extracted according to the established method (16). The gliadins, HMW-GSs and LMW-GSs were fractionated using reversed phase high-performance liquid chromatography (HPLC) described by Gao et al. (25). The SDS extractable and unextractable proteins (EPP and UPP) were separated by size-exclusion HPLC, as described by Gao et al. (25). The Glu/Gli ratio, H/L ratio and UPP% were measured using HPLC referred to the method reported by Li et al. (13), and the calculation formulas are as follows:


$$\begin{array}{lll} Glu/Gli\;ratio & = & \frac{Glutenin\;area}{Gliadin\;area}\times 100\%\\ \;H/L\;ratio & = & \frac{HMW-GS\;area}{LMW-GS\;area}\times 100\%\\ \;UPP\% & = & \frac{UPP\;area}{\left(EPP\;+\;UPP\right)\;area}\times 100\% \end{array}$$

The above parameters were based on the results with three independent replicates for each sample.

### Determination of Total Polyphenol Content (TPC) and Total Flavonoids Content (TFC)

The TPC in wheat and the four hulless barley samples was determined using Folin-Ciocalteau method as described by Xu et al. (10). The flour samples (1.5 g) were soaked in 70% methanol (15 mL) at room temperature for 2 h and then centrifuged at 6,000 × g for 15 min. The resultant supernatant (0.2 mL) was added to a 1 mL Folin-Ciocalteau chromogenic solution and 3 mL sodium carbonate solution, and then left out of light for 15 min. The absorbance value of the extracted sample was measured at 725 nm (Shimadzu UV-1800, Kyoto, Japan). The TPC was quantified using a standard gallic acid (Sangon Biotech Co., Ltd., Songjiang, Shanghai, China) curve (0–0.15 mg/mL). The results of total polyphenol content were expressed as mg of gallic acid equivalents (GAE)/g of flour.

The total flavonoids in wheat and the four hulless barley samples were extracted with 80% methanol at room temperature for 4 h according to the procedure of Gujral et al. (12). Then the mixture was centrifuged at 6,000 × g for 15 min. The supernatant (1 mL) was successively added with sodium nitrite (0.15 mL), aluminum nitrate (0.15 mL) and sodium hydroxide (2 mL), and then placed at room temperature for 15 min. The detection wavelength was 517 nm and the absorbance value of the samples was determined using a spectrophotometer (Shimadzu UV-1800, Kyoto, Japan). The TFC was quantified using a standard rutin (Sangon Biotech Co., Ltd., Songjiang, Shanghai, China) curve (0–0.1 mg/mL). The results were reported as mg of rutin equivalents (RE)/g of flour. The analyses for both TPC and TFC were conducted in three individual replicates for each sample.

### Determination of β-Glucan Contents

The content of β-glucan in wheat and the four hulless barley samples was measured according to the method reported by Moza and Gujral (5) using a β-Glucan (Mixed Linkage) Assay Kit (Megazyme International Ireland Ltd., Bray, Ireland). Each sample was measured in three independent replicates.

### Isolation and Number Distribution Analysis of Starch Granules

Starch was isolated from the wheat and the four hulless barley samples by manual washing as described by Li et al. (26). The grain flour was soaked in 0.2% sodium hydroxide solution for 24 h, and then filtered through a 100-mesh sieve. The filtrate was centrifuged at 4,000 × g for 20 min, and the supernatant was discarded and the uppermost yellow layer of the pellet was scraped off. The lower layer of the starch was washed with 75% ethanol for three times, and then dried in an oven at 40°C and passed through a 200-mesh sieve.

To measure the number distribution of starch granules, the Microtrac S3500 laser diffraction analyzer (Microtrac Inc., Pennsylvania, USA) was used as described by Cao et al. (19). The diameter of starch granules was not more than 10 μm was regarded as B-type starch. Each sample was measured three times.

### Determination of Apparent Amylose Content (AAC)

The AAC in wheat and the four hulless barley samples was measured using an Amylose/Amylopectin Assay Kit (Megazyme International Ireland Ltd., Bray, Ireland) (19). Each sample was measured in three individual replicates.

### Quantitative Analysis of Gluten Micro-Structure in the Formulated Doughs

The flour (2 g) of each sample was mixed and stained with 1.2 mL rhodamine B solution (0.01 mg/mL) to form a dough sample (17). The dough samples were placed at room temperature for 10 min to ensure the complete diffusion of the dye (17). After a small dough sample (2 × 2 mm) was placed on slide and flattened with cover-slip, micro-structure of dough was observed under a confocal laser scanning microscopy (CLSM) (Olympus, Tokyo, Japan), and five different images of each sample with a resolution of 512 × 512 pixel and a size of 211.5 × 211.5 μm (for 40× objective) were captured. The AngioTool64 (version 0.6a) was used to quantitatively analyze the images of each dough sample to calculate three gluten micro-structure parameters: protein area, total protein length and lacunarity (13).

### Fundamental Rheological Properties of Formulated Doughs

Dough rheological characteristics: storage modulus (G′) and loss modulus (G″) were determined by a rheometer (AR2000ex, TA Instruments, New Castle, USA) with a parallel plate geometry (40 mm in diameter, 1 mm in gap) following the previous report (13). The dough sample was obtained by manually mixing 2 g of flour with 1.3 mL of distilled water and rested for 20 min to relax any residual stresses. The test was carried out at a sweep frequency of 0.1–10 *Hz* and a strain of 0.1%. The tan δ was calculated as the ratio of G″ to G′. The mean value of each rheological parameter was calculated by three replicates.

### Determination of Mixing Properties of the Formulated Doughs

The mixing properties of the formulated doughs were measured by Mixolab 2 (Chopin, Paris, France) following the method described by Torbica et al. (27). The “Chopin +” protocol was used in the experiment, and the amount of flour and water was automatically calculated by the instrument. Mixolab curve of each dough sample was processed to determine the nine dough mixing parameters, including water absorption (WA), dough development time (DDT), dough stability time (DS), maximum dough consistency (C1), protein weakening (C2), starch gelatinization (C3), cooking stability (C4), final viscosity (C5), and gelatinization temperature (GT), as per the protocol of Graça et al. (28). Each sample was analyzed by three independent replicates.

### Bread Baking

Baking performance was analyzed according to Xu et al. (10). The flour (100 g), yeast (1 g), sugar (6 g), salt (1 g), and appropriate amount of water (added according to the water absorption of Chopin data) were stirred in a bread blender for 10 min to form uniform dough. Yeast, sugar, and salt were purchased from a retail store in Yangling, Shaanxi Province. Thereafter, the dough was fermented at 36°C for 2 h, and then baked at 210°C for 30 min. Three loaves of bread were prepared in one batch for each formulation. The bread was cooled to room temperature for further measurements.

### Specific Volume of Bread

Specific volume of bread samples was measured in triplicate according to Spina et al. (29). The volume of bread was determined using the rapeseed displacement method, and the weight of bread was also weighed. The specific volume of the bread was the ratio of the volume measured to the weight of the bread sample.

### Bread Texture

The textural properties of bread samples were analyzed using Texture Analyser (TVT6700, Perten, Huddinge, Sweden) according to previous methods (30) with some modifications. The bread was sliced (50 mm thick) with a slicing machine. The double cycle compression protocol was used in the experiment, and the sample was compressed with a cylindrical aluminum probe (a diameter of 25 mm) at the test speed of 1.7 mm/s. Firmness, cohesiveness, springiness and resilience were determined based on the texture profile analysis curves. The measurements were repeated thrice.

### Sensory Evaluation

The sensory evaluation of bread was analyzed according to the method described by Xu et al. (10) with minor modifications. Five volunteers took part in the evaluation of appearance, color, texture, taste and overall acceptability of bread on a nine-point category scale ranging from 1 (extremely weak) to 9 (extremely strong). Participants were required to gargle with water between the two samples and evaluate the next sample after an interval of 2 min to ensure that evaluation was not affected by the interaction between the samples.

### *In vitro* Starch Hydrolysis

Starch digestion properties of the bread samples were measured according to the model established by Toutounji et al. (31). Freshly baked bread (5 g) was accurately weighed in 50 mL centrifuge tube where 40 mL sodium acetate buffer (0.2 M, pH 6.0) was added. All samples were shaken at 200 rpm for 5 min and then equilibrated to 37°C. Then working enzyme solution (5 mL, 1 U/mL pancreatic α-amylase and 5 U/mL amyloglucosidase) was added and the mixture was stirred under 200 rpm for 3 h. Digesta samples (0.2 mL) were collected from each tube during the digestion phase at six time points (0, 30, 60, 90, 120, and 180 min), and immediately placed in a boiling water bath for 20 min to stop the enzymatic activity of α-amylase and amyloglucosidase, followed by centrifugation at 4,000 × g for 10 min. Finally, the glucose content in the supernatant was determined by D-Glucose Assay Kit (Megazyme International Ireland Ltd., Bray, Ireland). Each sample was measured in three individual replicates.

### Determination of X-Ray Diffraction (XRD) and Nutritional Ingredients

#### Preparation of Formulated Flour (Before Baking) and Bread Flour (After Baking)

The formulated flour (before baking) and bread (after baking) were freeze-dried for 48 h, then ground into powder and passed through a 200-mesh sieve for further analyses.

#### X-Ray Diffraction (XRD) Analysis

The XRD patterns of the starch was analyzed using a powder X-ray diffractometer (D8 ADVANCE A25, Bruker, Germany) according to the method reported by Cao et al. (19). The samples were analyzed at a high voltage of 40 kV and a current of 40 mA. The scanning range of diffraction intensities was 5°-40° (2θ angle range) at a speed of 0.019°.

#### Determination of TPC and TFC

The TPC and TFC in formulated flours and bread flours were determined by the method described in section Determination of Total Polyphenol Content (TPC) and Total Flavonoids Content (TFC).

#### Determination β-Glucan Contents

The content of β-glucan in formulated flours and bread flours was determined by the method described in section Determination of β-glucan Contents.

### Statistical Analysis

Analysis of variance (ANOVA) was carried out using SPSS v. 22.0 (SPSS Inc., Chicago, IL, USA), and least significant differences (LSD) test was used to analyze the differences between samples with a confidence level of 95% (*P* < 0.05).

## Results and Discussion

### Chemical Components of the Wheat Variety and the Four Hulless Barley Varieties

Chemical components of the wheat variety and the four hulless barley varieties were determined (Table 1). JM had slightly higher protein content than the four hulless barley varieties did. Interestingly, the Glu/Gli ratio of the four hulless barley varieties was higher than that of JM. Given that the ratio of Glu/Gli is a major indicator that reflects the rheological behavior of dough (13), the variations of Glu/Gli ratio in wheat and hulless barley may have different contributions to dough rheological properties. The HPLC analyses show that JM has higher H/L ratio and UPP% than the four hulless barley varieties do, which may be attributed to lower gluten protein content in hulless barley. The Glu/Gli ratio, H/L ratio, and UPP% are conventional important indices to evaluate the quality of wheat (13, 15). To date, there are still no universal standards for the quality of hulless barley. Therefore, this study can provide useful information for measuring the Glu/Gli ratio, H/L ratio, and UPP% in hulless barley in the future.

**Table 1:** Parameters related to grain quality, protein component and bioactive ingredient of wheat and four hulless barley varieties.

**Sample**	**Grain quality determined by near-infrared reflectance**				**Protein component**			**Bioactive ingredient**		**β-glucan content (%)**	**B-type starch granule content (%)**	**Apparent amylose content (%)**
	**Moisture (%)**	**Protein content (%)**	**Total starch content (%)**	**Total dietary fiber content (%)**	**Glu/Gli ratio (%)**	**H/L ratio (%)**	**UPP%**	**Total polyphenol content (mg·g** ^**−1**^ **)**	**Total flavonoids content (mg·g** ^**−1**^ **)**			
JM	10.60 ± 0.04b	16.62 ± 0.06a	64.89 ± 0.47a	2.01 ± 0.15c	62.24 ± 1.52c	49.30 ± 1.25a	43.60 ± 0.23a	0.57 ± 0.09d	0.34 ± 0.03e	0.55 ± 0.07c	71.59 ± 0.85a	23.78 ± 2.21b
HB1	5.80 ± 0.30d	15.27 ± 0.30b	47.93 ± 0.07d	13.17 ± 0.19a	63.98 ± 4.01c	24.89 ± 0.06c	31.55 ± 0.85b	1.02 ± 0.11c	1.05 ± 0.10d	3.60 ± 0.14ab	47.29 ± 0.6d	32.63 ± 2.30a
HB2	13.44 ± 0.05a	12.76 ± 0.06c	60.98 ± 0.15a	12.09 ± 1.17ab	84.80 ± 0.61b	42.72 ± 0.78b	25.44 ± 0.74c	2.26 ± 0.18a	1.80 ± 0.15c	3.97 ± 0.04a	64.54 ± 0.90b	28.45 ± 2.53a
HB3	12.99 ± 0.05a	11.54 ± 0.32d	55.36 ± 0.62b	11.38 ± 0.06ab	87.42 ± 2.78b	48.81 ± 2.45a	25.42 ± 0.77c	1.59 ± 0.13b	2.44 ± 0.06b	3.68 ± 0.31ab	57.30 ± 1.65c	36.39 ± 4.47a
HB4	8.79 ± 0.31c	12.67 ± 0.24c	52.67 ± 0.52c	10.71 ± 0.68b	99.63 ± 3.69a	43.25 ± 0.40b	18.91 ± 0.81d	2.19 ± 0.16a	3.10 ± 0.12a	3.27 ± 0.02b	72.42 ± 2.78a	27.33 ± 3.04a

*Different lower-case letters in the same column indicate significant difference (P < 0.05)*.

*H/L ratio, high- to low-molecular-weight glutenin ratio; UPP (%), the percentage of unextractable polymeric protein to total polymeric protein; Glu/Gli (%), the ratio of gluten to gliadin content*.

As shown in Table 1, the total starch content of hulless barley is significantly lower than that of JM, which falls in the previously reported range of starch content (45.7–66.4 %) in the 112 Chinese hulless barley genotypes with variable starch characteristics (32). There are significant differences in total starch content, B-type starch content and apparent amylose content among the JM and the four hulless barley samples. Except for HB4 with high B-type starch content close to JM, the other three hulless barley samples show lower B-type starch content than JM does. A recent study has shown that the content of B-type starch is positively correlated with dough stability (14), and thereby it is speculated that the HB4 formulated dough was more stable. In addition, the apparent amylose content of the four hulless barley varieties was slightly higher than that of JM. It has been reported that foods with high amylose content have relatively low starch digestion (23). Thus, the starch digestibility of hulless barley formulated bread is expected to be desirable.

The content of nutritional ingredients of wheat and hulless barley varieties was further analyzed (Table 1). The results show that the TPC of the four hulless barley varieties ranges from 1.02 to 2.26 mg·g^−1^, which is significantly higher than that of JM (0.57 mg·g^−1^). As expected, the TFC of the four hulless barley varieties (1.05–3.10 mg·g^−1^) was also comparatively higher than that of JM (0.34 mg·g^−1^). The TPC and TFC in hulless barley have been reported to have a range of 2.71–4.37 mg·g^−1^ and 0.56–2.86 mg·g^−1^, respectively (5, 6). These results are slightly different from the previous ones, with lower TPC but higher TFC, which may be attributable to variations among varieties or different extraction methods. The content of total dietary fiber and β-glucan in hulless barley flour ranges from 10.71 to 13.17% and from 3.27 to 3.97%, respectively, which is significantly higher than that in wheat flour. Taken together, the component analysis further confirmed that hulless barley in the current study was rich in nutritional ingredients compared with wheat. Consequently, the nutritional quality of the formulated bread should be effectively improved by adding appropriate amount of hulless barley flour.

### Micro-Structure of Gluten in Formulated Dough System

**Figure 1:**
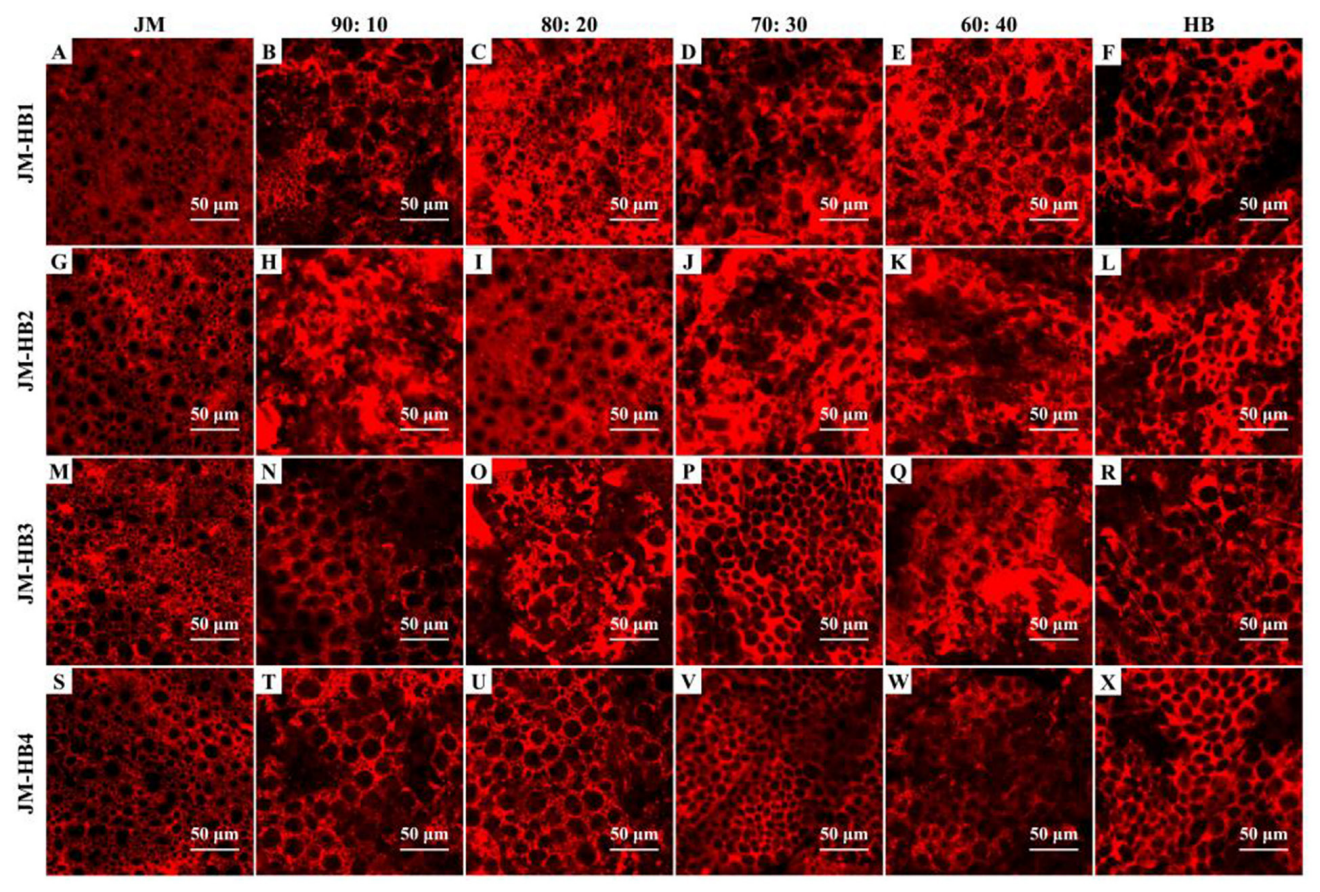
Micro-structure of gluten network observed by confocal laser scanning microscopy (CLSM). The formulated dough samples were prepared with one super-strong gluten wheat variety (JM) and four hulless barley varieties (HB1, HB2, HB3, and HB4) with different ratios, and stained with Rhodamine B. Scale bar = 50 μm.

**Figure 2:**
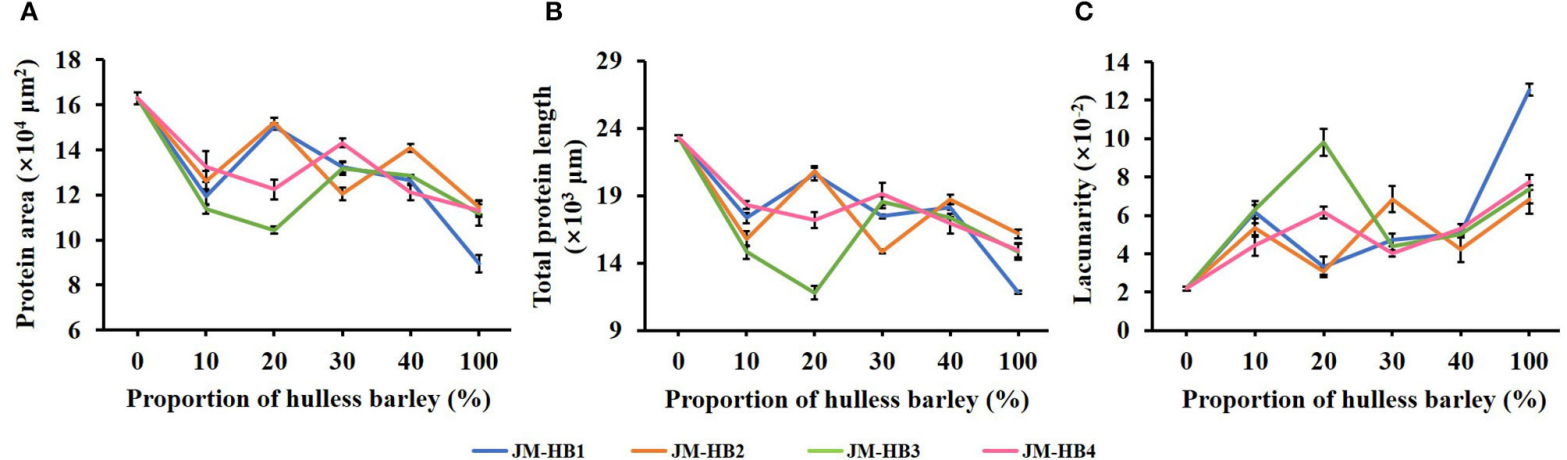
Quantitative analysis of the gluten network in hulless barley formulated dough samples determined by AngioTool software. **(A)** Protein area. **(B)** Total protein length. **(C)** Lacunarity.

In order to determine an optimum ratio of hulless barley flour to wheat flour and to measure the impact of the hulless barley addition on gluten structure, the formulated dough system was characterized by CLSM. It can be observed that JM flour, as a flour sample with very strong gluten, displays a quite continuous and dense gluten network structure, while the gluten skeleton of the formulated dough shows more disruption, indicating the result of hulless barley supplementation (Figure 1). The parameters (protein area, total protein length, and lacunarity) characterizing gluten micro-structure were calculated to illustrate changes of gluten network along with increase of hulless barley addition in the dough system (Figure 2). The addition of hulless barley as wheat substitute led to a significant decrease in protein area and total protein length (Figures 2A,B), while opposite trend was observed for lacunarity (Figure 2C). This result can be attributed to the dilution of wheat gluten protein due to supplementation with hulless barley which has less polymeric protein (Table 1); in addition, more dietary fiber brought by hulless barley flour may have more interaction with gluten protein, which prevented gluten protein from binding with water (10), and resulted in the larger voids in gluten network observed in Figure 1. Since lacunarity measures the void size distribution which reflects the uniformity of gluten network (17), strong dough has low lacunarity, highly developed reticular structure, small void size, and regularity (15). In addition, it was also found that lacunarity is related to the stability time of wheat dough (14). The results showed that addition of hulless barley disrupted micro-structure of gluten and destabilized dough. At different addition proportions, JM-HB1-20, JM-HB2-20, JM-HB3-30, and JM-HB4-30 exhibit higher protein area, longer total protein length, and smaller lacunarity (Figure 2). Previous studies have reported that adding an appropriate proportion of potato pulp can enhance the gluten network structure (18); similarly, these results show that the gluten structure of the formulated systems was not gradually destroyed when increasing proportion of hulless barley. Therefore, it is necessary to further analyze the dough rheological properties of the formulations, which helps to shed light on breeding the suitable hulless barley variety and find an optimal flour formula.

### Fundamental Rheological Properties of Dough

**Figure 3:**
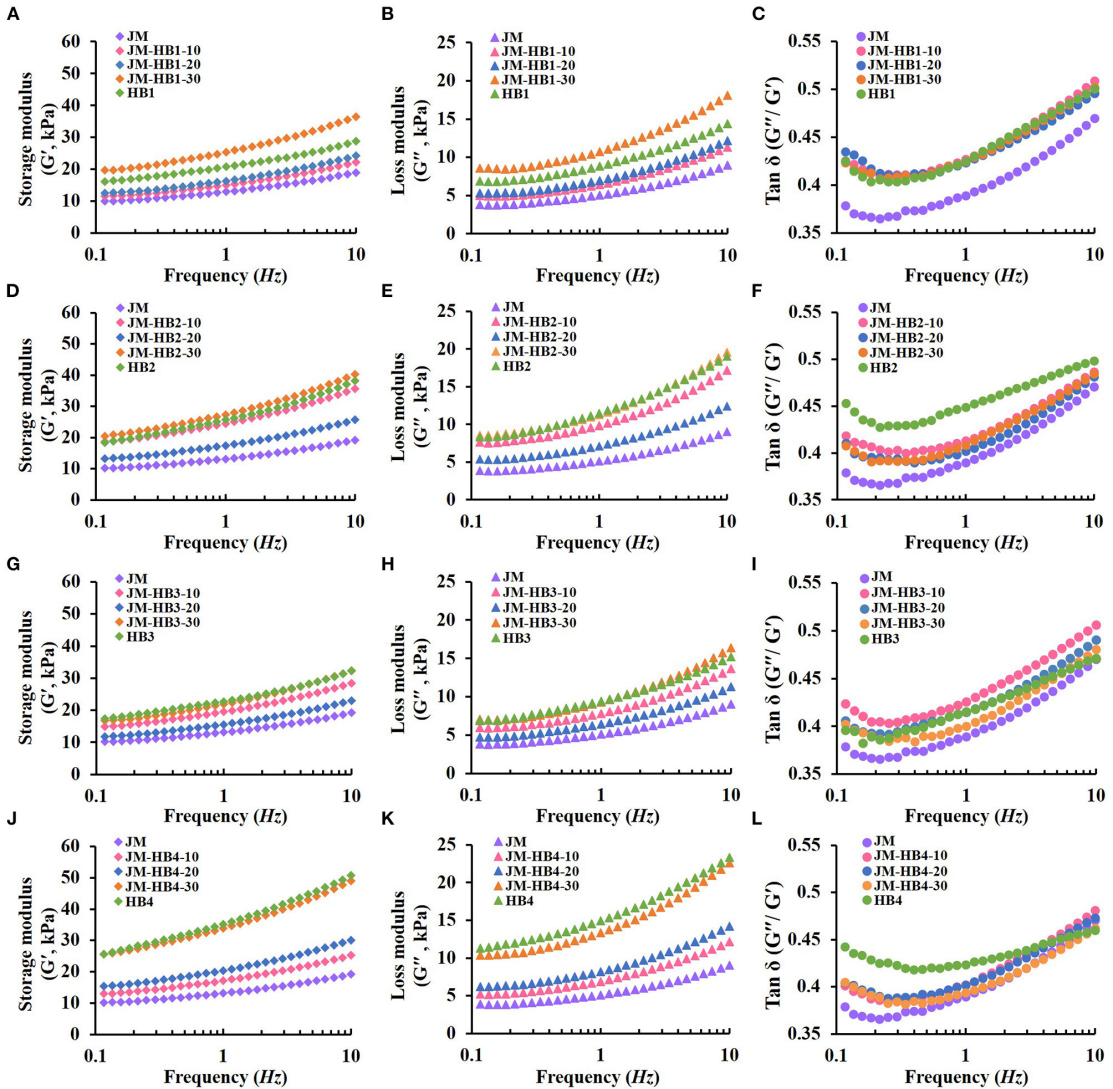
Viscoelastic properties of hulless barley formulated dough. **(A,D,G,J)**: Storage modulus (G′); **(B,E,H,K)**: Loss modulus (G^′′^); **(C,F,I,L)**: Tan δ (G^′′^/G′).

After the above gluten structure analyses, the dough systems supplemented with hulless barley flours at <40% substitution were further analyzed for their rheological properties. Dynamic measurements of dough rheological properties dough provide information about viscoelasticity, which is of great significance to food production. The changes of G′, G″ and tan δ of dough samples are shown in Figure 3. The G′ relates to dough's ability to store energy elastically; while the loss modulus G′ represents the dough's ability to dissipate stress and reflects the viscosity of the dough (13). In the whole process, G′ is larger than G″, indicating that elasticity is dominant (33). After adding hulless barley flour, changes in viscoelasticity of dough were more pronounced. Compared with JM dough, the addition of hulless barley significantly increased G′ and G″. In addition, G′ and G″ of dough exhibited different trends after adding different hulless barley varieties, which may be attributed to the different cross-linking levels and structural changes in gluten network (Figure 1). The tan δ represents the ratio of viscosity to elasticity of dough. A lower tan δ value indicates that dough has a more elastic structure (33). With the increase of frequency, the tan δ value of dough decreased initially and then increased, indicating that the elasticity of dough was not dominant at high frequency. Among the four hulless barley varieties, the tan δ value of HB3 was the lowest (Figure 3I). This can be attributed to differences in flour components i.e., protein and starch among the different hulless barley varieties (Table 1) (4, 11). In previous studies, it has been found that gluten, starch and their interactions all affect the rheological properties of dough (13, 14, 19, 34). Therefore, the possible reasons for the changes in rheological properties of the hulless barley formulated dough can be explained by two things: addition of hulless barley diluted the wheat gluten protein structures and when the hulless barley flour was added, new interactions between starch and protein can be formed in the formulated dough system, which impacts dough behavior. It is worth noting that the tan δ value of HB2 and HB4 formulated dough basically coincides with that of JM dough (Figures 3F,L), which indicates the great dilution resistance of JM. Therefore, anti-dilution wheat varieties such as JM are suggested to be used as base flour for bread formulations. Selection of hulless barley varieties is important for the production of formulated products as well. Given that the components of hulless barley flours from different varieties may affect the properties of the formulated dough differently, it is reasonable to further study differences in composition of gluten and starch of the four hulless barley varieties (i.e., JM-HB1-20, JM-HB2-20, JM-HB3-30, and JM-HB4-30).

### Dough Mixing Properties

The parameters lacunarity, G′, G″ and tan δ are significantly related to dough stability time which relates to the quality of final product (14, 33). Therefore, based on the results of the micro-structure of gluten and the rheological properties of the formulated dough system, the optimal substitution levels of each hulless barley variety closest to JM were determined for further analysis of their dough mixing properties. Mixolab curves show that addition of hulless barley flours has different effects on gluten and starch properties, compared with the control dough (Supplementary Figure S1). In the first 8 min, the curves of JM-HB3-30 and JM show similar trends and from the 15th min to the end JM-HB2-20 and JM-HB4-30's curves are similar to JM's, which indicates they had similar starch characteristics. This can be attributed to the differences in protein structure and starch physicochemical properties among different hulless barley varieties (4, 11). Dough mixing parameters reflect the effect of the added hulless barley on gluten strength and starch gelatinization (Table 2). For water absorption, there were no significant differences among the wheat dough and formulated dough. Previous studies demonstrated that barley bran rather than wheat bran increases water absorption of dough (12), which suggests hulless barley flour is more suitable as supplement for bread formulations. The DDT and DS of the hulless barley supplemented doughs were significantly lower than those of JM wheat dough, indicating that the supplementation of hulless barley flours decreased dough strength to some degree, but still at an acceptable level for bread-making. Reduced dough strength can be attributed to addition of hulless barley flours. Similar results were observed by Liu et al. (35), who added potato flour into wheat flour for steamed bread. This phenomenon can be explained by the fact that hulless barley and potato flours have high content of fiber (5, 35), which compete for moisture with gluten or starch, resulting in reduction of cross-linkage among gluten proteins and dough stability time (36). However, different from potato pulp, hulless barley flour contains polymeric proteins, which are expected to contribute substantially to the formation of gluten structure in the formulated dough system, even though UPP% in hulless barley flour is not as much as that in wheat flour (Table 1). Thus, hulless barley can be considered as a good supplement for bread making with regard to the functionality of gluten components.

**Table 2:** Dough mixing properties of wheat supplemented with different amounts of hulless barley flours.

**Sample**	**WA (%)**	**DDT (min)**	**DS (min)**	**C1 (Nm)**	**C2 (Nm)**	**C3 (Nm)**	**C4 (Nm)**	**C5 (Nm)**	**GT (**°**C)**
JM	65.45 ± 0.75a	8.96 ± 0.19a	10.89 ± 0.19a	1.17 ± 0.01a	0.61 ± 0.01a	1.73 ± 0.02a	1.64 ± 0.05a	2.11 ± 0.39a	77.05 ± 0.85ab
JM-HB1-20	66.10 ± 0.10a	6.47 ± 0.39b	8.22 ± 0.05c	1.06 ± 0.01c	0.36 ± 0d	1.27 ± 0.01c	0.17 ± 0.01d	0.23 ± 0.01c	75.30 ± 0.70b
JM-HB2-20	65.95 ± 0.05a	6.34 ± 0.14b	8.45 ± 0.10bc	1.06 ± 0c	0.47 ± 0.01c	1.72 ± 0.01a	1.40 ± 0.02b	1.95 ± 0.05ab	78.85 ± 0.15a
JM-HB3-30	66.60 ± 0.20a	4.88 ± 0c	8.68 ± 0.09bc	1.10 ± 0b	0.47 ± 0c	1.63 ± 0.01b	0.86 ± 0.01c	1.23 ± 0.02b	76.80 ± 0.10b
JM-HB4-30	66.20 ± 0.10a	5.95 ± 0.67c	8.73 ± 0.18b	1.09 ± 0.01bc	0.51 ± 0b	1.76 ± 0.01a	1.44 ± 0.01b	1.77 ± 0.27ab	78.40 ± 0.20ab

*Different letters within the same column are statistically different (P < 0.05)*.

*WA, water absorption; DDT, dough development time; DS, dough stability time; C1, dough development; C2, protein weakening; C3, starch gelatinization; C4, cooking stability; C5, final viscosity; GT, gelatinization temperature*.

After mixing dough for 8 min, the torque value decreases with increase of dough temperature (Supplementary Figure S1), indicating denaturation of protein in dough (12). The results show that protein weakening (C2) of the formulated dough was significantly lower than that of JM dough, which may be due to the dilution of gluten protein (12). During starch gelatinization (C3), peak torque was observed at 1.73 Nm for the JM dough and in the range of 1.27–1.76 Nm for the formulated doughs. No significant change was observed in starch gelatinization between JM-HB2-20 and JM-HB4-30 dough while a big drop was seen in JM-HB1-20 and JM-HB3-30, compared to JM dough. The results indicate that HB1 and HB3 had more expansion resistance in the process of heating gelatinization. This finding is consistent with a previous study reporting that supplementation of β-glucan can decrease viscosity of dough, resulting in a lower peak viscosity of reconstituted dough (37). After starch gelatinization, cooking stability (C4), and final viscosity (C5) of the formulated doughs were significantly lower than those of JM dough. These results show that addition of hulless barley flour had a negative effect on gelatinization of starch. This can be explained by the fact that high level of dietary fiber in a formulated dough system demands more available water (22), and insufficient hydration would suppress starch swelling. Another potential reason would be that β-glucan chains may surround starch granules and bond with moisture, which is otherwise absorbed by the starch granules, thereby reduce peak viscosity and final viscosity of starch (37). Therefore, abundant β-glucan in hulless barley flour may also be one of the reasons affecting gelatinization of starch.

### Textural Properties and Sensory Evaluation of the Formulated Bread

**Figure 4:**
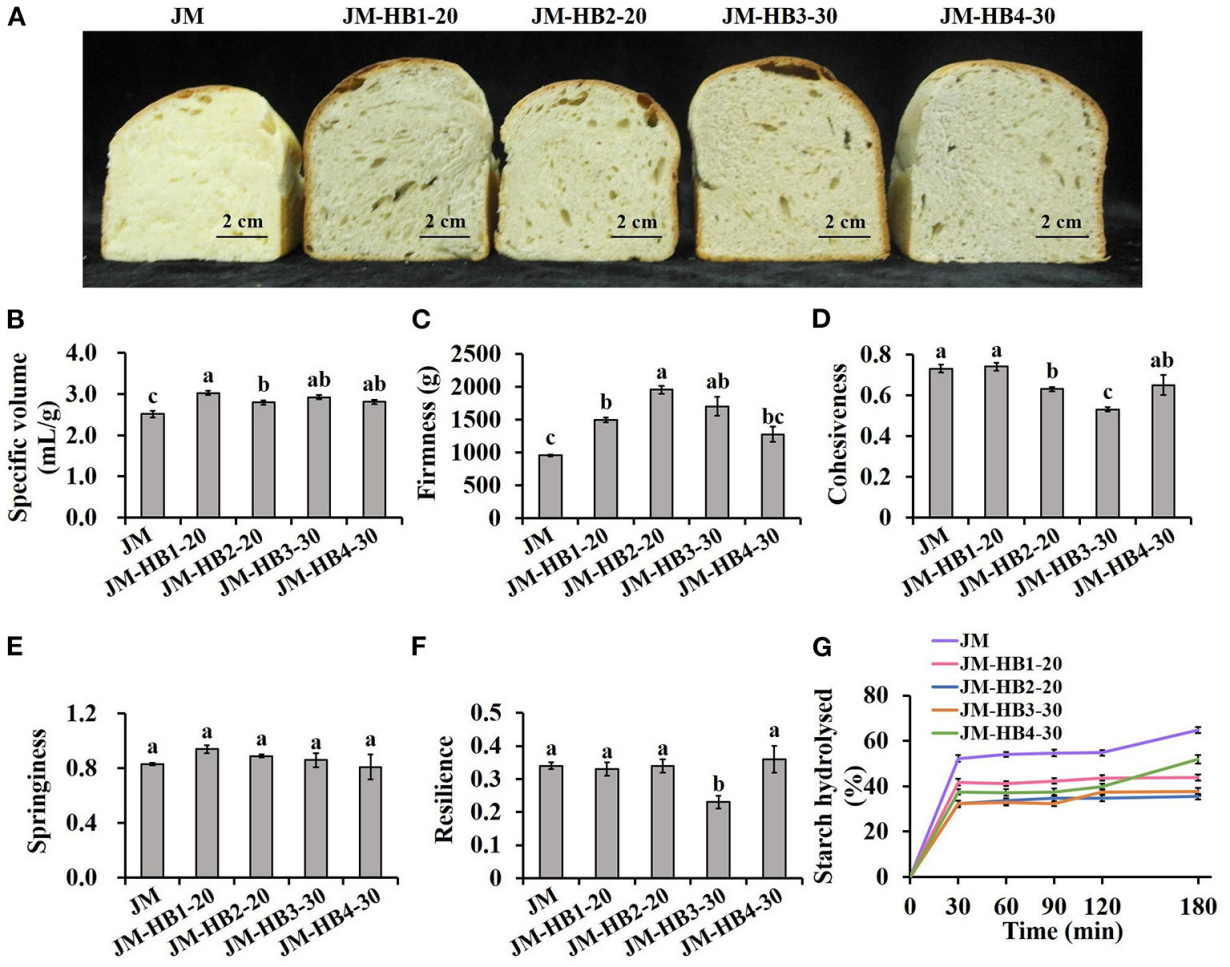
The baking performance **(A,B)**, textural properties **(C–F)** and starch digestibility **(G)** of hulless barley formulated bread. Different letters above the columns for specific volume, firmness, cohesiveness, springiness, and resilience indicate significant difference at *P* < 0.05.

Specific volume of bread is closely related to its baking performance, and also a visual attribute of consumers' choice (10). As shown in Figures 4A,B, JM-HB1-20, JM-HB2-20, JM-HB3-30, and JM-HB4-30 display significantly larger specific volume than JM does, indicating that the formulated bread are visually more preferable. In addition, the structure of bread crumb is also an important feature to evaluate the quality of bread (30). The crumb structure of the formulated breads was rougher than that of JM bread. Among them, the JM-HB4-30 bread displays similar uniform and smooth crumb structure as the JM bread does, except for a few irregular bubbles formed during baking (Figure 4A). Furthermore, the textural parameters (firmness, cohesiveness, springiness, and resilience) of the formulated bread were compared (Figures 4C–F). The results showed that there was no significant difference in firmness, cohesiveness, springiness, and resilience between JM-HB4-30 and JM breads, whereas JM-HB3-30 bread had lower resilience and cohesiveness than JM did. Springiness is commonly related to resilience, and decrease in springiness and resilience is suggested to associate with loss of crumb elasticity (30). Cohesiveness represents internal resistance of bread structure, and low cohesiveness indicates that crumbs are vulnerable to outer force or stress (10). The above results indicate that among the four formulated bread samples, JM-HB4-30 has most desirable bread texture as a whole.

Sensory analysis of bread showed that the scores of appearance, color and texture of JM-HB1-20, JM-HB2-20, JM-HB3-30, and JM-HB4-30 breads were significantly lower than those of JM bread (Table 3), which may be due to richer fiber content and more colored substances in the seed coat of hulless barley, which led to poor structure and color of the formulated breads. However, the four formulated breads had significantly better taste than JM bread, and there was no significant difference in overall acceptability between JM-HB4-30 and JM breads.

**Table 3:** Sensory quality of the supplemented with different amounts of hulless barley flours.

**Sample**	**Appearance**	**Color**	**Texture**	**Taste**	**Acceptability**
JM	7.8 ± 0.1a	8.7 ± 0.1a	8.1 ± 0.3a	6.3 ± 0.9a	8.6 ± 0.1a
JM-HB1-20	5.6 ± 0.1d	5.8 ± 0.1c	4.8 ± 0.6b	6.8 ± 1.4a	7.0 ± 0.2bc
JM-HB2-20	7.1 ± 0.1b	7.3 ± 0.1b	5.4 ± 0.6b	7.7 ± 0.4a	7.6 ± 0.1b
JM-HB3-30	7.0 ± 0.1bc	6.0 ± 0.2c	6.3 ± 0.9ab	8.1 ± 0.4a	6.9 ± 0.3c
JM-HB4-30	6.6 ± 0.4c	7.1 ± 0.5b	5.1 ± 0.3b	6.7 ± 1.4a	8.0 ± 0.1ab

*Different lower-case letters in the same column show significant difference among samples (P < 0.05)*.

### *In vitro* Starch Digestibility and Crystalline Structure of the Formulated Bread

A simulation model of intestinal digestion was used to study *in vitro* starch digestibility of the formulated breads (Figure 4G). The results show that all bread samples have similar patterns in starch hydrolysis, in which the content of hydrolysed starch increased rapidly in the first 30 min of digestion, and then gradually reached equilibrium. Compared with the JM wheat bread, the levels of hydrolysed starch in the formulated breads are significantly lower, indicating that the formulated breads with hulless barley flours had lower starch digestibility, and is potentially beneficial for controlling blood glucose after meal (10). These results can be attributed to the differences in amylose content among wheat and various hulless barley flours and be the fact that the amylose content was higher (23). Because when starch is heated and then cooled, amylose can interact with other molecules quickly to form complexities that resist digestion, while amylopectin molecules recombine slowly and are easier to be digested (38). This difference explains why food products with high amylose content have lower starch digestibility. Moreover, high content of β-glucan in hulless barley flours (Table 1) is also responsible for the desired starch digestibility of the formulated breads, which has been reported in the previous study (5). Bread made from wheat flours reconstituted with barley rich in β-glucan has a lower content of rapidly digestible starch, and thus its consumption can increase intestinal viscosity and thus reduce hydrolysed starch (39).

**Figure 5:**
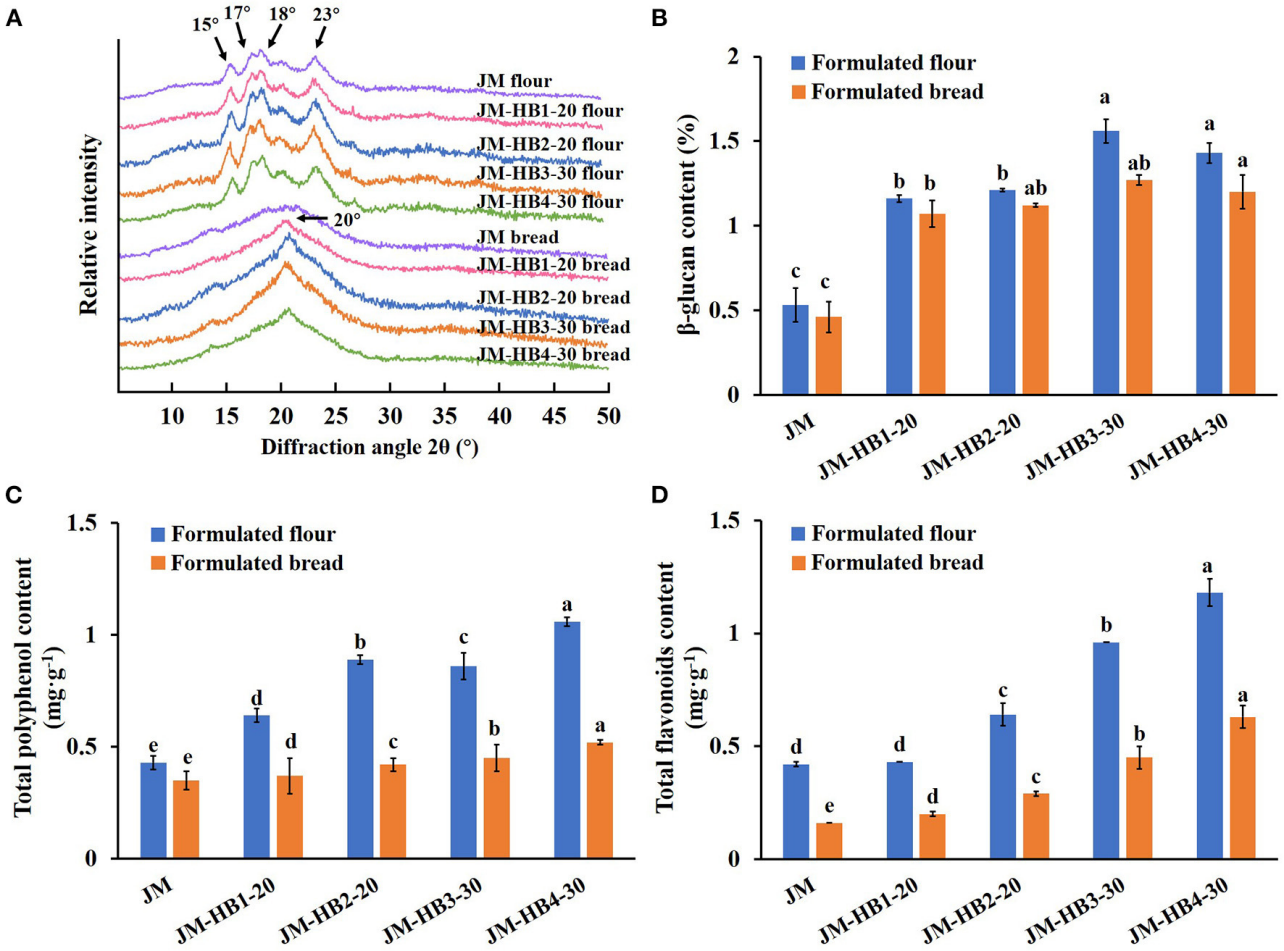
The crystalline structure **(A)** and nutritional ingredients **(B–D)** of the formulated flours and breads. Different letters above the columns for β-glucan content, total polyphenol content, and total flavonoids content indicate significant difference at *P* < 0.05.

Furthermore, crystalline structure of starch plays a decisive role in starch digestibility (40). To reveal decreased starch hydrolysis, it is necessary to further investigate changes in the crystalline structure of the formulated breads. XRD analysis indicates that the formulated flours showed a typical A-type crystalline structure of cereal starch, with strong diffraction peaks at 2θ of 15, 17, 18, and 23° (Figure 5A). Two extra peaks located at 2θ of 13 and 20° were also observed (Figure 5A) in the X-ray diffraction pattern, which changed to a V_7_-hydrate form upon extrusion cooking of starch, indicating that amylose-lipid or starch-protein-lipid complexes were formed in the formulated breads (40). This starch-lipid complex, formed by fatty acids occupying the cavities of single amylase helices, was stable, which can resist the hydrolysis of amylase (20, 40). Compared with JM, intensity and area of diffraction peak at 20° were significantly increased, and crystallinity of JM-HB2-20 and JM-HB3-30 starch in bread was particularly higher. Consistent with the characteristics of the above digestive systems, hulless barley increased the amylose content of the formulated breads, and the resultant higher amylose content tended to form V-type structure with fatty acids and resulted in increased resistance of starch to digestion (20, 40).

Besides, the bread formulated with hulless barley contains more nutritional and functional components. Although there was some loss during baking, the levels of β-glucan, polyphenols and flavonoids in formulated breads were still significantly higher than those in JM bread (Figures 5B–D). Calculation shows that the levels of β-glucan, polyphenols and flavonoids in JM-HB4-30 bread were, respectively, increased by 160.87, 48.57, and 293.75%, compared with those of JM bread. Given that daily consumption of 3 g β-glucan can effectively reduce cholesterol levels in the body (41), the bread formulated with hulless barley in the current study has high nutritional value, which should greatly promote human health. Therefore, it is suggested that the formulated bread should serve as a new health food to enrich consumers' diet.

## Conclusion

The study demonstrated that even though addition of hulless barley flour to wheat flour has a negative effect on gluten micro-structure and dough behavior, the formulated breads possessed enhanced specific volume, improved textural features, and lower digestibility. Moreover, the taste and overall acceptability of JM-HB4-30 bread were not significantly different from those of JM bread, and its nutritional components (β-glucan, polyphenols and flavonoids) were significantly increased. These results indicate that proper formulations with selected hulless barley varieties can produce functional bread with desirable starch digestibility and still acceptable baking quality, which is suitable for diabetic treatment. In the future for the purpose of production of hulless barley-supplemented food, more attention should be paid to selection of hulless barley and wheat varieties and substitution level. The results of this study have a guiding role in the development and utilization of hulless barley for functional bread in food industry.

## Data Availability Statement

The raw data supporting the conclusions of this article will be made available by the authors, without undue reservation.

## Author Contributions

LY: investigation, formal analysis, and writing—original draft, review, and editing. YM: methodology, data curation, and writing—review and editing. YZ, YP, RT, and LG: investigation and data curation. XY, YY, and ZW: resources. KW, XC, and XG: supervision, project administration, and writing—review and editing. All authors contributed to the article and approved the submitted version.

## Funding

This work was supported by Open Project of State Key Laboratory of Plateau Ecology and Agriculture, Qinghai University [grant number 2020-KF-003], the Agriculture Research System of China [grant number CARS-05], the National Key Research and Development Program of China [grant number 2020YFD1001403], Taishan Industrial Experts Programme [grant number LJNY202006], Key Research and Development Program of Shandong Province (Major Science and Technology Innovation Project) [grant number 2020CXGC010805] and Agricultural Scientific and Technological Innovation Project of Shandong Academy of Agricultural Sciences [grant number CXGC2021A02].

## Conflict of Interest

The authors declare that the research was conducted in the absence of any commercial or financial relationships that could be construed as a potential conflict of interest.

## Publisher's Note

All claims expressed in this article are solely those of the authors and do not necessarily represent those of their affiliated organizations, or those of the publisher, the editors and the reviewers. Any product that may be evaluated in this article, or claim that may be made by its manufacturer, is not guaranteed or endorsed by the publisher.

## Correction Note

A correction has been made to this article. Details can be found at: 10.3389/fnut.2026.1849997.

## Supplementary Material

Supplementary Figure S1 Supplementary Figure S1 The mixing profiles of hulless barley formulated dough samples.
