# Correction: Effect of hulless barley flours on dough rheological properties, Baking Quality, and Starch Digestibility of Wheat Bread

**DOI:** 10.3389/fnut.2026.1849997

**Published:** 2026-05-06

**Authors:** Liwei Yu, Yanrong Ma, Yiyue Zhao, Yilin Pan, Renmei Tian, Xiaohua Yao, Youhua Yao, Xinyou Cao, La Geng, Zhonghua Wang, Kunlun Wu, Xin Gao

**Affiliations:** 1 State Key Laboratory of Plateau Ecology and Agronomy, Qinghai Key Laboratory of Hulless Barley Genetics and Breeding, Qinghai Subcenter of National Hulless Barley Improvement, Qinghai University, Xining, China; 2 State Key Laboratory of Crop Stress Biology in Arid Areas and College of Agronomy, Northwest A&F University, Xianyang, China; 3 Shandong Academy of Agricultural Sciences/National Engineering Laboratory for Wheat and Maize/Key Laboratory of Wheat Biology and Genetic Improvement in North Yellow and Huai River Valley, Crop Research Institute, Ministry of Agriculture, Jinan, China; 4 Institute of Crop Science, Zhejiang University, Hangzhou, China

**Keywords:** hulless barley, wheat bread, nutritional function, *in vitro* digestibility, dough mixing properties

There was a mistake in Figure 1 as published. Two of the JM sample images in Figure 1 exhibit partial rotational overlap. The corrected Figure 1 appears below.

**Figure 1:**
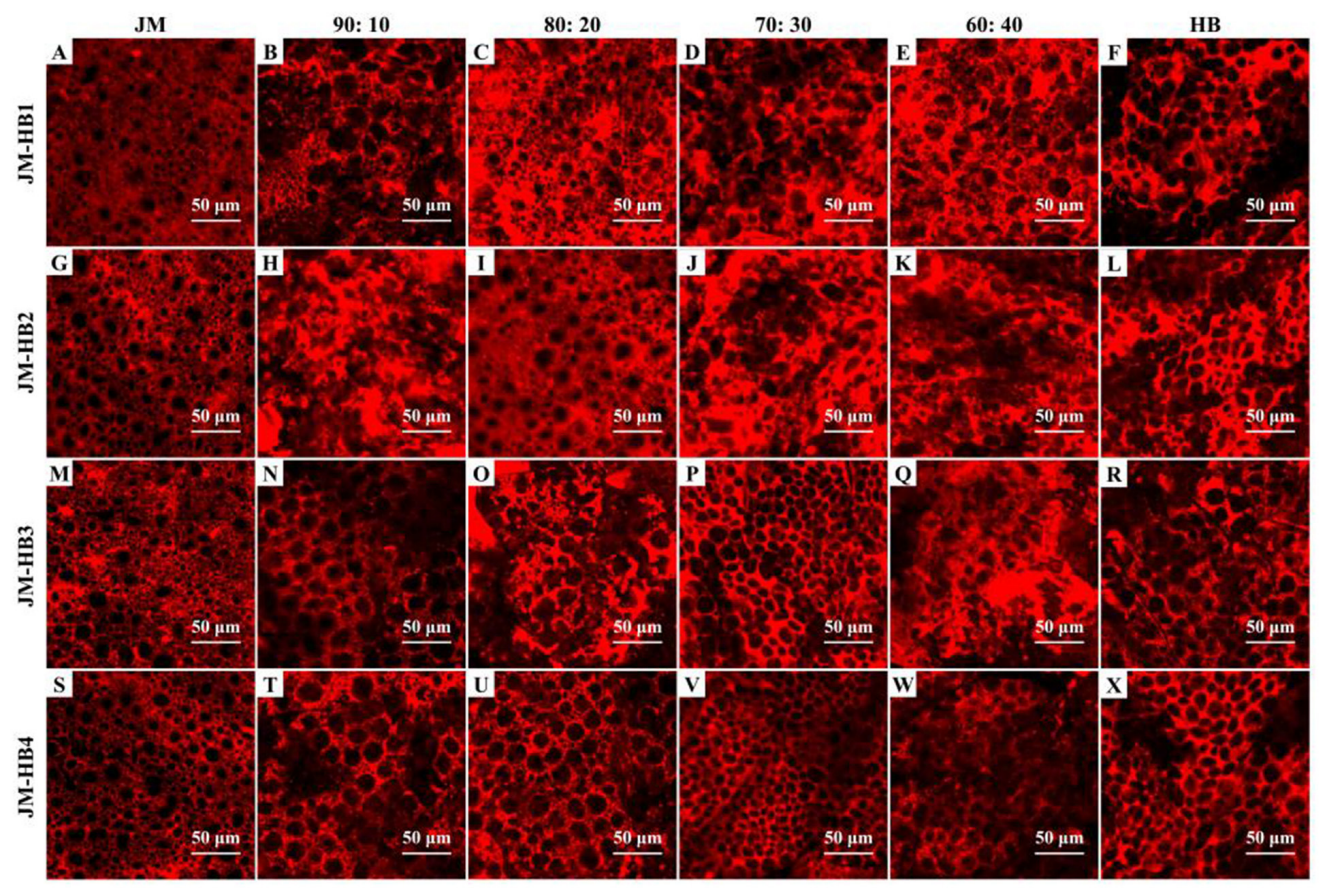
Micro-structure of gluten network observed by confocal laser scanning microscopy (CLSM). The formulated dough samples were prepared with one super-strong gluten wheat variety (JM) and four hulless barley varieties (HB1, HB2, HB3, and HB4) with different ratios, and stained with Rhodamine B. Scale bar = 50 μm.

The original version of this article has been updated.

