# Supplementary figures and images for: Effect of Hulless Barley Flours on Dough Rheological Properties, Baking Quality, and Starch Digestibility of Wheat Bread
Source: Front Nutr. 2021 Dec 13;8:785847. doi: 10.3389/fnut.2021.785847 (PMC8710734; doi:10.3389/fnut.2021.785847)

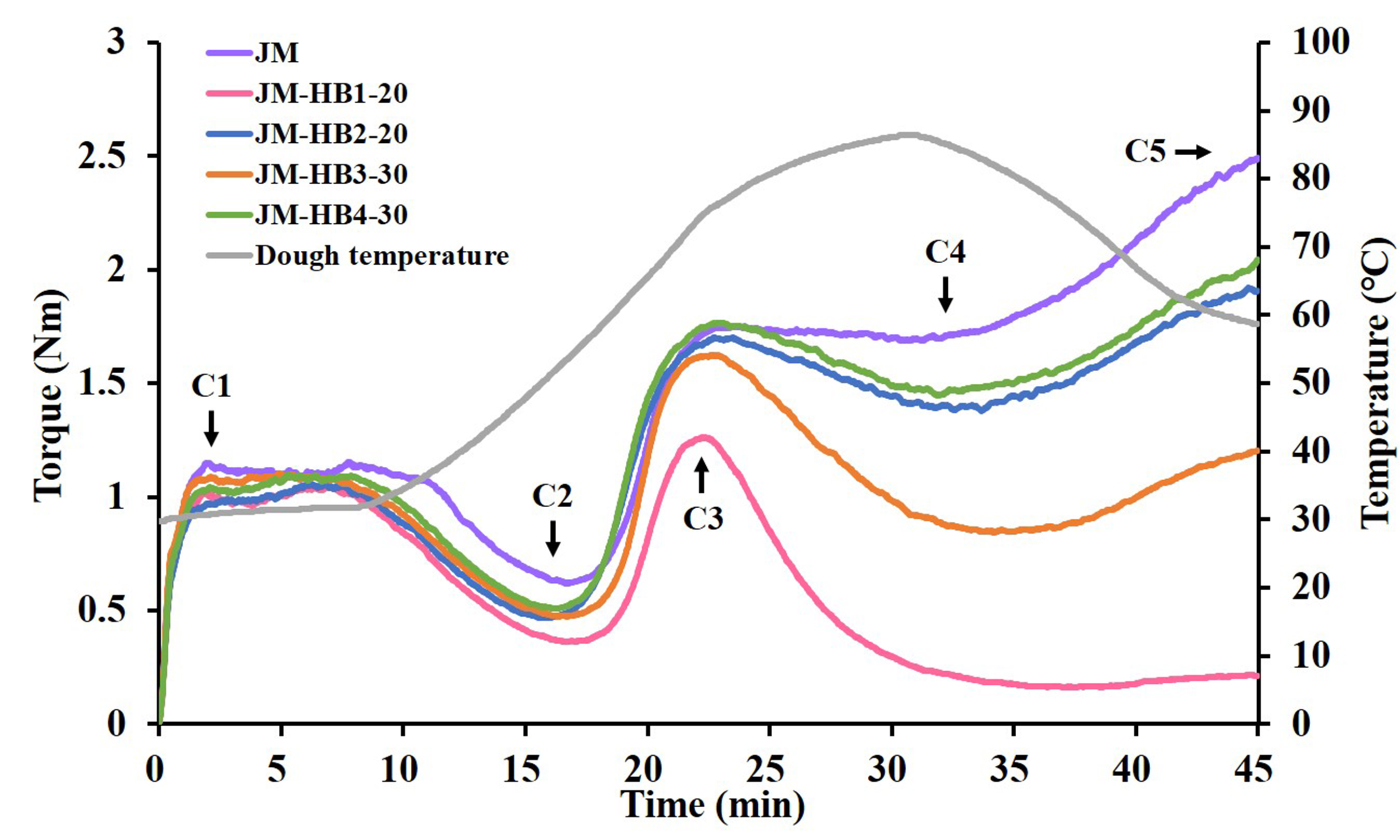

Supplement: Supplementary Figure S1 — The mixing profiles of hulless barley formulated dough samples. [file Image_1.JPEG]
